# Comparison of Macular Thickness in Diabetic Macular Edema Using Spectral-Domain Optical Coherence Tomography and Time-Domain Optical Coherence Tomography

**DOI:** 10.1155/2012/959721

**Published:** 2012-01-18

**Authors:** Masashi Kakinoki, Taichirou Miyake, Osamu Sawada, Tomoko Sawada, Hajime Kawamura, Masahito Ohji

**Affiliations:** Department of Ophthalmology, Shiga University of Medical Science, Seta-Tsukinowa-cho, 520-2192 Otsu, Japan

## Abstract

*Purpose*. To compare the macular thicknesses in diabetic macular edema (DME) measured with spectral-domain optical coherence tomography (SD-OCT) and time-domain (TD) OCT. *Patients and Methods.* The average macular thicknesses of 50 eyes of 29 patients with DME were measured using SD-OCT and TD-OCT. *Results.* The mean macular thicknesses measured with TD-OCT and SD-OCT were 401.5 ± 117.8 *μ*m (mean ± SD) and 446.2 ± 123.5 *μ*m, respectively. The macular thicknesses measured with the two devices were well correlated (Pearson's product moment correlation, *r* = 0.977, *P* < 0.001). A significant correlation was found between the best-corrected visual acuity and the retinal thickness measured by TD-OCT and SD-OCT (Pearson's product moment correlation, TD-OCT, *r* = 0.34; *P* < 0.05; SD-OCT, *r* = 0.32; *P* < 0.05). *Discussion.* The mean macular thickness measured with SD-OCT was about 45 *μ*m thicker than that measured with TD-OCT. Attention should be paid when comparing data obtained using different OCT machines.

## 1. Introduction

Optical coherence tomography (OCT), which provides B-mode retinal images, has become essential for diagnosing retinal disease and glaucoma [1–9] since the technology was first reported by Huang et al. in 1991 [10]. OCT also provides quantitative retinal thickness data, which are useful to monitor retinal changes in clinical and research settings [3].

Time-domain OCT (TD-OCT) includes an interferometer that measures the echo delay time of light that is reflected and backscattered from various retinal microstructures. The echo time delays of light beam reflected from the retinal microstructure are compared with the echo time delays of the same light beam reflected from a reference mirror at known distances. The TD method samples only one point at a time. Therefore, it takes a relatively long time to obtain A-scan and B-mode retinal images, and it is almost impossible to obtain a three-dimensional retinal image.

In spectral-domain OCT (SD-OCT), light beams returning from the sample and reference paths are combined at the detector, a spectrometer that resolves the interference signals throughout the depth of each A-scan without varying the length of the reference path. This is possible because the spectrometer resolves the relative amplitudes and phases of the spectral components backscattered from all depths of each A-scan simultaneously using the Fourier transformation. This allows SD-OCT to acquire retinal images about 50 times faster compared with TD-OCT [11]. The substantial increase in scan speed allows acquisition of three-dimensional data sets.

A few studies have compared the macular thicknesses of patients with diabetic macular edema (DME) obtained with TD-OCT and SD-OCT [12, 13]. We compared the retinal thickness measurements obtained with the two OCT devices in subjects with DME to understand the differences in measurements between the two OCTs. We already reported the difference in mean retinal thickness between TD-OCT and SD-OCT in normal eyes [14]. To the best of our knowledge, the current study is the first to compare the macular thickness measurements from the two OCT devices between normal subjects and patients with DME. 

## 2. Patients and Methods

The macular thickness was measured in 50 eyes of 29 patients with DME using TD-OCT (Stratus OCT, Carl Zeiss Meditec, Dublin, CA, USA) and SD-OCT (Cirrus HD-OCT, Carl Zeiss Meditec, Dublin, CA, USA) to determine a correlation between the devices. The mean patient age was 68.0 ± 9.0 years (range, 45–85 years). Of the 29 subjects with DME, 18 were men and 11 were women.

**Figure 1:**
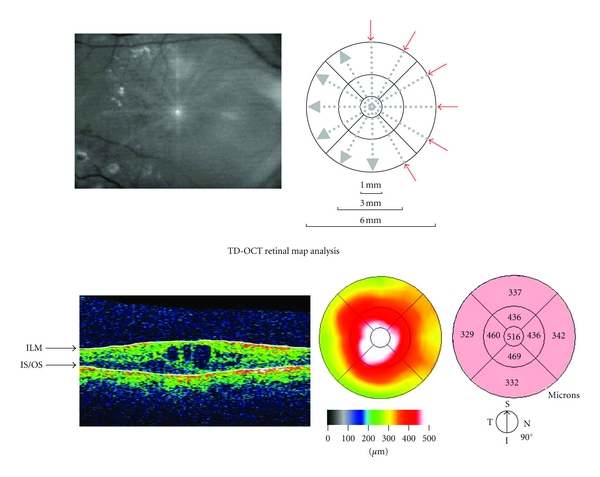
(Top) Fast macular thickness scan pattern with TD-OCT. (Bottom) The mean retinal thickness at the central 1 mm circle is 516 *μ*m in a 74-year-old man.

With TD-OCT, the macular thickness data were obtained using the fast macular thickness scan pattern (Figure 1). This scan pattern acquires six linear B-scans in a continuous, automated sequence. The scans are centered at the fovea in a radial pattern and separated by 30-degree increments. Each B-scan consists of 128 A-scans. The axial resolution of TD-OCT is less than 10 *μ*m according to the manufacturer's data.

**Figure 2:**
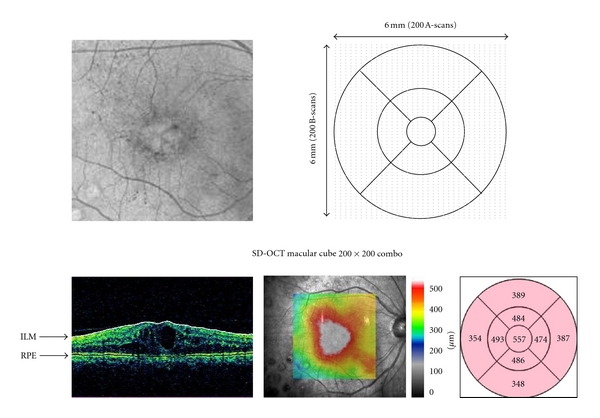
(Top) Macular cube 200 × 200 mode with SD-OCT. (Bottom) The mean retinal thickness at the central 1 mm circle is 557 *μ*m in the same 74-year-old man.

The Macular Cube 200 × 200 scan pattern in SD-OCT generates a data cube through a 6 mm square grid by acquiring a series of 200 horizontal scan lines, each comprised of 200 A-scans (Figure 2) with an axial resolution of 5 *μ*m.

The average retinal thickness at the central 1 mm area was analyzed with both OCT machines. About 128 points were measured within a 1 mm circle with TD-OCT and about 872 points with SD-OCT.

Three operators measured the macular thickness of each subject using the two OCT instruments on the same day. Apparent segmentation failures in TD-OCT and SD-OCT were excluded from this study.

The best-corrected visual acuities (BCVAs) were obtained in decimal VA and converted to logarithm of the minimum angle of resolution (log MAR) for statistical analysis.

## 3. Results

**Figure 3:**
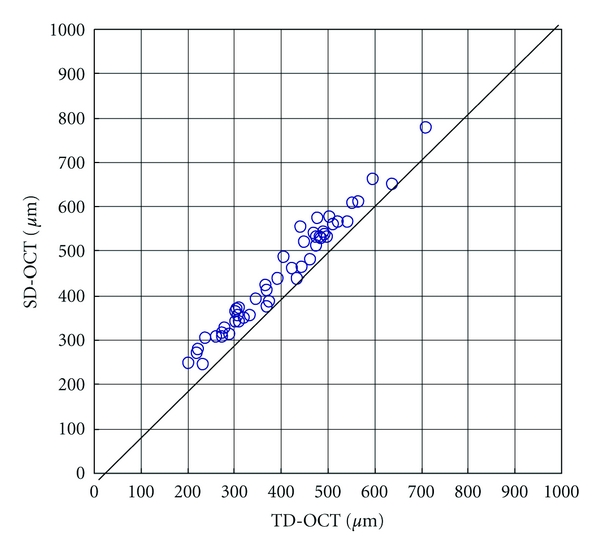
The difference between TD-OCT and SD-OCT average retinal thicknesses in patients with DME in a central 1 mm area is about 45 *μ*m. These two data sets are well correlated (correlation coefficient, 0.977, *P* < 0.001, Pearson's product moment correlation).

The mean macular thicknesses in patients with DME measured with TD-OCT was 401.5 ± 117.8 *μ*m (mean + SD; range, 203–712 *μ*m) and with SD-OCT 446.2 ± 123.5 *μ*m; range, 245–775 *μ*m). The mean macular thickness with SD-OCT was 44.7 *μ*m thicker than that with TD-OCT, which was a significant difference (*P* < 0.001, paired *t*-test). The macular thickness measured with TD-OCT correlated well with that measured with SD-OCT (Pearson's product moment correlation,  *r* = 0.977,  *P* < 0.001) (Figure 3).

**Figure 4:**
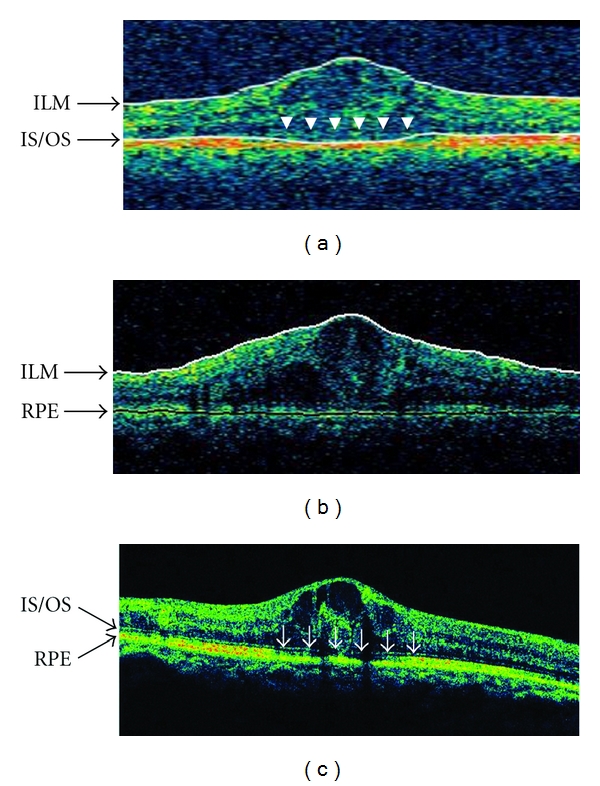
The ILM and IS/OS are traced by a white line using TD-OCT, although the true IS/OS line is thought to be that indicated by the arrowheads in the retinal thickness mode (a). The ILM and RPE are traced by a white and black line by the macular thickness mode of SD-OCT (b). The IS/OS line is indicated by a vague line in the five-line mode (arrowheads) (c).

A representative case was that of a 75-year-old man with DME. TD-OCT traced the ILM and IS/OS line automatically, and the central retinal thickness was 543 *μ*m (Figure 4(a)). SD-OCT traced the ILM and RPE automatically, and the central retinal thickness was 567 *μ*m (Figure 4(b)), which was 24 *μ*m thicker than the TD-OCT measurement. Compared with the five-line mode of the SD-OCT (Figure 4(c)), the true IS/OS line of the TD-OCT may be the pale line that is defined by the arrowheads (Figure 4(a)). If TD-OCT automatically traced the true IS/OS line, the central retinal thickness would be thinner than 543 *μ*m. We assumed that the retinal thickness using TD-OCT was thicker than the actual retinal thickness, and as a result, the difference in the retinal thicknesses between the two machines was small. This is one reason that the macular thickness in normal subjects is about 15 *μ*m thicker than in patients with DME.

**Figure 5:**
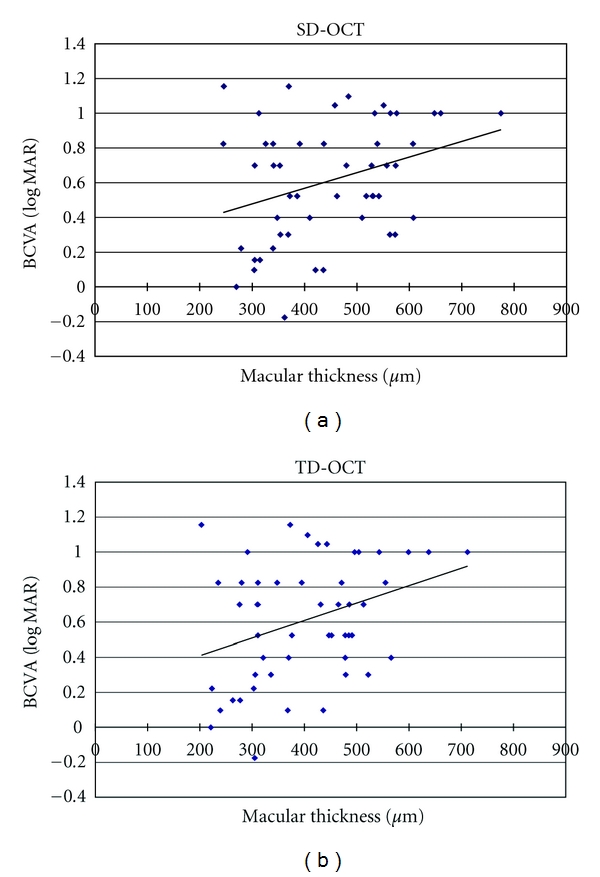
There is a significant correlation between TD-OCT (b) and SD-OCT (a) relationship for the BCVA and the retinal thickness (Pearson's product moment correlation, TD-OCT, *r* = 0.34, *P* < 0.05; SD-OCT, *r* = 0.32, *P* < 0.05).

The relationship between the BCVA and the retinal thickness also was evaluated, and a significant correlation was found between the two devices (Pearson's product moment correlation, TD-OCT, *r* = 0.34; *P* < 0.05; SD-OCT, *r* = 0.32; *P* < 0.05) (Figure 5).

## 4. Discussion

In the current study, the average retinal thickness measured with TD-OCT was 401.5 ± 117.8 *μ*m and with SD-OCT 446.2 ± 123.5 *μ*m, a difference of about 45 *μ*m. The difference in the average retinal thicknesses between the devices appears to have resulted from the different definitions of the retinal thicknesses. TD-OCT defines retinal thickness as the distance from the surface of the inner limiting membrane (ILM) to the boundary between the inner and outer segments of the photoreceptors (IS/OS). SD-OCT defines the retinal thickness as the distance from the surface of the ILM to the surface of the retinal pigment epithelium (RPE). The different algorisms explain the different results. Lammer et al. compared the retinal thickness of DME between TD-OCT and SD-OCT and reported mean difference was 58.5 *μ*m [12]. Forooghian et al. also reported that the mean difference of the two machines was 53.0 *μ*m [15]. These results were almost same as our result, and they suggested that different algorisms make the different results. Attention should be paid to the retinal thickness when data from different machines are compared, although the retinal thickness in patients with DME measured by SD-OCT correlated strongly with that measured by TD-OCT.

We reported previously that when using SD-OCT in normal subjects, the macula was 60 *μ*m thicker than when measured with TD-OCT [14]. In the current study, when using SD-OCT in subjects with DME, the macula was 45 *μ*m thicker than when measured with TD-OCT. The difference in the macular thickness between TD-OCT and SD-OCT in normal subjects was about 15 *μ*m thicker than that in patients with DME. With TD-OCT, the fast macular thickness scan pattern acquires six linear B-scans in a continuous automated sequence. The scans are centered at the fovea in a radial pattern and separated by 30-degree increments. With SD-OCT, the Macular Cube 200 × 200 scan pattern generates a data cube through a 6 mm square grid by acquiring a series of 200 horizontal scan lines, each comprised of 200 A-scans. The average retinal thickness at the central 1 mm circle was about 128 points with TD-OCT and about 872 points with SD-OCT. Compared with TD-OCT, the higher reliability of SD-OCT is based on the uniformity and the larger number of scan points. The scan pattern and the scan accuracy may explain the difference in the macular thickness between normal subjects and DME.

Using TD-OCT, the IS/OS line disappeared in some areas or was not clearly detected in some cases. The measurement line was not traced on IS/OS and traced on the RPE line. This may result in a thicker measurement than the actual thickness on TD-OCT.

As in previous studies, we also found a correlation between the macular thickness and BCVA in subjects with DME [16, 17]. The Diabetic Retinopathy Clinical Research Network reported that the relationship between the VA and central retinal thickness measured by OCT was linear [16]. However, Koleva-Georgieva and Sivkova reported no correlation between the BCVA and retinal thickness [18], which may have resulted from the small number of subjects and that six of nine eyes had macular ischemia. The low VA could be due to the serous macular detachment with large cystoid spaces and the presence of macular ischemia.

In conclusion, the mean retinal thickness in patients with DME measured with SD-OCT was about 45 *μ*m thicker than that with TD-OCT. Care should be taken when comparing retinal thicknesses between the two OCT machines.

##  Conflict of Interests

The authors have no proprietary interest in any aspect of this paper.
